# Exploring the usefulness of Lexis diagrams for quality improvement

**DOI:** 10.1186/s12911-019-1017-3

**Published:** 2020-01-08

**Authors:** Sara Dahlin

**Affiliations:** Technology Management and Economics, Chalmers University of Technology, 412 96 Gothenburg, Sweden

**Keywords:** Quality improvement, Survival data, Feedback, Timeliness, Complexity, Lexis diagram

## Abstract

**Background:**

Visualization is important to aid practitioners in understanding local care processes and drive quality improvement (QI). Important aspects include timely feedback and ability to plot data over time. Moreover, the complexity of care also needs to be understood, as it affects the variation of care processes. However, there is a lack of QI methods visualizing multiple, related factors such as diagnosis date, death date, and cause of death to unravel their complexity, which is necessary to understand processes related to survival data. Lexis diagrams visualize individual patient processes as lines and mark additional factors such as key events. This study explores the potential of Lexis diagrams to support QI through survival data analysis, focusing on feedback, timeliness, and complexity, in a gynecological cancer setting in Sweden.

**Methods:**

Lexis diagrams were produced based on data from a gynecological cancer quality registry (4481 patients). The usefulness of Lexis diagrams was explored through iterative data identification and analysis through semi-structured dialogues between the researcher and domain experts (clinically active care process owners) during five meetings. Visualizations were produced and adapted by the researcher between meetings, based on the dialogues, to ensure clinical relevance, resulting in three relevant types of visualizations.

**Results:**

Domain experts identified different uses depending on diagnosis group and data visualization. Key results include timely feedback through close-to-real-time visualizations, supporting discussion and understanding of trends and hypothesis-building. Visualization of care process complexity facilitated evaluation of given care. Combined visualization of individual and population levels increased patient focus and may possibly also function to motivate practitioners and management.

**Conclusion:**

Lexis diagrams can aid understanding of survival data, triggering important dialogues between care givers and supporting care quality improvement and new perspectives, and can therefore complement survival curves in quality improvement.

## Background

The aim of quality improvement (QI) is “to make changes that will lead to better patient outcomes (health), better systems performance (care) and better professional development (learning)” ( [1], p., 2). Improvement efforts should be guided by data, which can enable practitioners to understand how their local processes vary and take relevant action on that basis [2, 3]. As process variation is constantly affected by a wide range of factors, both within and outside of the practitioners’ control [3], feedback gained by following data over time is needed to enable an understanding of the process [4], preferably in real time [5]. Data visualization enables analysis and sensemaking by domain experts based on their knowledge and experience [6, 7]. They identify and observe datapoints, discover patterns, and compare the results to their prior understanding, drawing inferences and building hypotheses [8]. This may answer questions such as “Are we on track?” “Is something negative happening that we need to address?” “or even “Is the process improving?” Such exploration may later be followed by confirmatory statistical analysis [9]. Data visualization also has the purpose of communicating findings [10], and has been used as a basis for discussion between stakeholders for QI [11]. Performance-related feedback has also been shown to be important for motivating staff, regardless of whether it indicates a need for improvement or not [12].

**Fig. 1:**
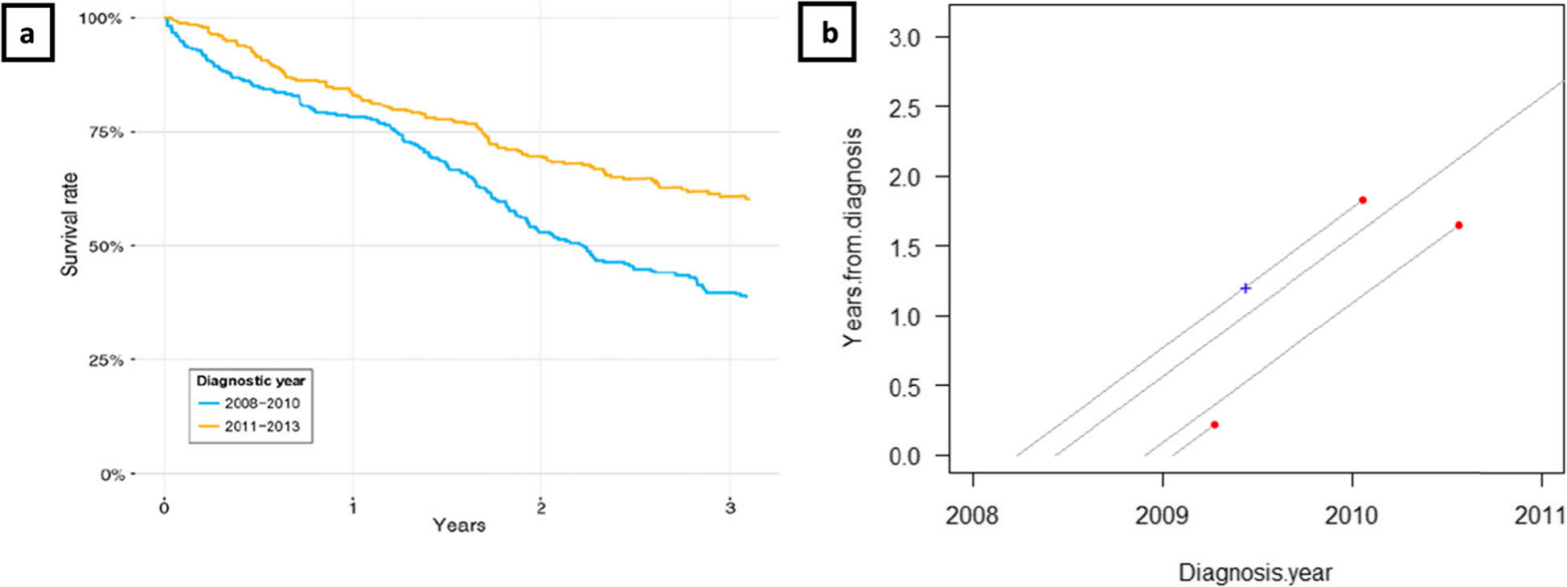
Examples of different visualizations. 1**a**. Survival curve comparing two cohorts. Reprinted from Dahm-Kähler et al. [15], with permission from Elsevier. 1**b**. Lexis diagram, presenting data of four hypothetical patients, each patient represented by a lifeline starting on diagnosis date (y = 0) and continuing upwards until patient’s death, or extending to right border if patient is alive at data extraction. The lifelines’ 45-degree angle reflects equal time passing along both time axes in the Lexis diagram. Dots represent time of death, and crosses show other events occurring between diagnosis and death

To enable local understanding, methods visualizing local process change are used in several healthcare settings (see e.g. [13]). However, one area in which improvements could be made in the visual representation of data for QI purposes is survival data analysis. Such data are often analyzed through survival curves and used for evaluating improvement efforts (see e.g. [14]), but survival curves have another purpose; they contribute to “global knowledge” useful in (e.g.) medical research such as clinical trials of new treatments, or, as in Dahm-Kähler et al. [15], showing that centralization of surgery improves survival on a population level. Kaplan–Meier analysis is the most popular type of survival data analysis [16], resulting in a survival curve plot (exemplified in Fig. 1a) featuring time since an event on the x-axis and a survival probability measure on the y-axis, such as survival rate [15] or cumulative survival [16], presenting the estimated survival rate of (e.g.) a patient group with a particular diagnosis over a certain time interval since the starting event. For further information about Kaplan–Meier curves, see Jager et al. [16]. Its purpose is thus not to support local process understanding and action for QI and due to the need for large sample sizes and aggregation of data, other methods are needed to ensure timeliness and gaining feedback by following data over time, contributing to local improvements.

To follow local changes over time related to (e.g.) cancer care processes, survival data documented in health information systems may be usefully input into methods supporting understanding of local process changes.

Survival data typically include several important factors, such as time of diagnosis, time of death and cause of death, which may need to be understood simultaneously, as they together reflect care process complexity. Notably, the level of complexity in a (care) system depends on the number of components and their interrelatedness [17], and non-linearity also characterizes complex system behavior [18]; studying relevant interrelatedness between components within the care system may support understanding of the system [17] and thus aid local improvement action [2, 3]. Characterizing components’ interrelatedness becomes necessary to unravel the system’s complexity [17], and domain experts may in this way be supported in their understanding of local care process complexity and thereby guided into QI efforts [19]. To analyze such data, focus need to be set on understanding and visualizing the complexity of several process-related factors for feedback purposes. Specifically, as noted by Jiang et al. [20], combined visualization of aggregated, population-based data and patient-level raw data can powerfully reveal relevant patterns. QI literature gives limited attention to methods visualizing multiple process-related factors on an individual level over time and in a timely manner. Graphical excellence, being “that which gives to the viewer the greatest number of ideas in the shortest time with the least ink in the smallest space…telling the truth about the data” ( [21], p., 51), may be useful to guide the choice of method in this case, to bring domain experts effective feedback on their process. Through their domain expertise, they can make sense of data lacking statistical significance, and thereby build understanding which may support them in action.

Consequently, new methods addressing feedback (through following data over time) and visualizing complexity while also ensuring timeliness are preferred in the analysis of survival data for QI. Lexis diagrams is one such possible method, used to identify joint effects of age, period, and cohorts [22–24], and serve as a possible complement to subsequent statistical survival analysis [25, 26]. In its basic configuration, a Lexis diagram visualizes individual lifelines along two time axes: calendar time and age or year since diagnosis [27–29], where the lines extends in a 45-degree angle as time passes along both x and y axes. Figure 1b shows a basic Lexis diagram with hypothetical patients.

Lines and markings can be differently colored or shaped to represent different attributes [30, 31], for instance, line colors representing diagnoses; marking shapes representing care process events, and marking colors representing event attributes. New data (lines/patients or markings) can be continuously added, supporting timeliness in analysis. Lexis diagrams can for example be analyzed by counting markings, such as dots [30] or lines, across a section of the diagram [32], to identify patterns. Together, this versatile visualization may reveal the complex pattern of events, time between events and attribute data, that may enhance understanding [33]. Kaplan–Meier survival curves and Lexis diagram are compared in Table 1.

**Table 1:** Comparison between Kaplan–Meier curves and Lexis diagram

Method	Data	Process changes	Level	Data variables	Statistical analysis	Data set size
Kaplan–Meier	Aggregated data	Before- and after	Global	Single variable	Yes	Large
Lexis diagram	Raw data	Followed over time	Local	Multiple variables	No	Small

Lexis diagrams have been applied in several ways, such as for advanced graphical display of individual life histories [30] and population-level mortality dynamics [34]. With potential to elicit feedback by (e.g.) presenting trends in data [35], ensure timeliness through real-time monitoring [32], and visualize complexity through the use of different colors, markings, and time axes [33], they may help practitioners understand care given and improve actionable survival data analysis. However, despite the epidemiology–QI link [36], and the use of Lexis diagrams to understand survival epidemiologically (see e.g. [37, 38, 39]), few studies have considered Lexis diagrams as a QI aid (see for example [32]).

As the combination of Lexis diagrams, survival data, and support for QI seems still unaddressed, this study explores the potential of Lexis diagrams to support QI through survival data analysis, focusing on feedback, timeliness, and complexity.

## Method

### Case study

#### Context

The study context is gynecological cancer in Western Sweden. Survival data analysis is highly relevant to cancer diagnoses, since most cancer types are still life-threatening, and patients may be saved by improved care. This diagnosis group also received additional attention through the “Cancer Moonshot” program launched in 2016, through which quality improvement and learning healthcare systems contribute via continuous monitoring of clinical practice [40]. “Cancer Moonshot” aims to progress precision medicine—personalized care based on characteristics such as genetic differences [40]. With increased complexity of care, potentially resulting in small patient sample sizes [41], visualization through Lexis diagrams in combination with domain expert understanding could support cancer care quality improvement.

In this study, gynecological cancer data are used. Gynecological cancer diagnoses strike about 2800 women yearly in Sweden, with relatively low mortality, as noted, except for ovarian cancer. In Sweden, national quality registries for gynecological cancer are handled by Regional Cancer Centre West (RCC West). The ovarian cancer registry started in 2008, the uterine corpus cancer registry in 2010 and the cervical cancer registry in 2011. Designated process owners, who are clinically active physicians with great practical knowledge, are “owning” the process by being responsible for developing the care pathway for a specific cancer diagnosis, making them invaluable for quality improvement collaborations involving method assessment and development.

Reasons for choosing gynecological cancer are several. First, process owners are engaged and interested in collaboration regarding new methods. Second, gynecological cancer sub-diagnoses (ovarian, cervical, corpus) exhibit low incidence and/or high survival, expected to render small data sets, well suited for Lexis diagram [30]. Third, the three diagnosis subgroups have different care processes and patient characteristics, yielding a broader picture of Lexis diagram usefulness. Fourth, the gynecology cancer quality registry in Western Sweden is estimated 100% complete, minimizing risk of bias from missing data.

#### Research process

**Fig. 2:**
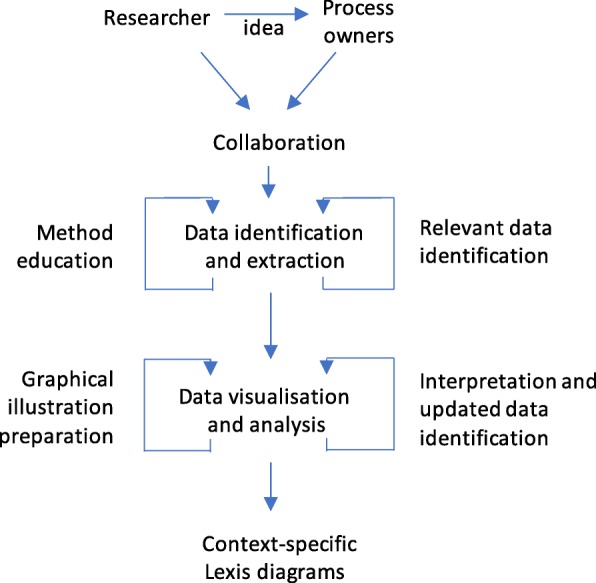
The collaborative research process with the researcher’s role on the left and process owner’s role on the right

The research process is presented in Fig. 2 below; it is similar to how Street et al. [42] addressed method development in a collaborative environment.

#### Setting the collaboration

Exploration of data for QI purposes demands good understanding of the visualization by those involved; therefore, a simple graphic display is preferred [4, 43]. Too much information may be difficult to grasp, so only relevant data should be included, to keep the illustration as simple as possible [21]. Thus, a collaboration entailing both domain expertise and method knowledge was necessary. The collaboration entailed the researchers and the four regional process owners for gynecological cancer in Western Sweden, being active physicians (three surgeons and one oncologist). The process owners contributed with medical and local data expertise to ensure relevant visualization and avoid misinterpretations. The researcher presented the methodological ideas to the process owners and supported them with methodological knowledge during method evaluation.

#### Data identification and extraction

Iterative data identification and analysis was performed through semi-structured dialogues during five meetings including the researcher and 1–4 process owners depending on availability. At one meeting, the statistician at RCC West, responsible for data extraction, was present to help answer questions regarding relevant variables. Meetings lasted 60–150 min each and were audio-recorded.

Relevant data and subgroups were identified by process owners and extracted from the Swedish quality register for gynecological cancer (sub-registers ovarian cancer, cervical cancer, corpus cancer) over the full length of the cancer registries (diagnosis date from 2008-01-01 for ovarian cancer , 2011-01-01 for cervical cancer, and 2010-01-01 for corpus cancer until 2016-12-31 for all diagnoses). Patients were followed until death or until the data extraction day (2018-11-17). Additional factors included surgery date, relapse date, and cause of death. Stratification of data was based on sub-diagnosis group and tumor severity, the main reasons for differences in care processes, such as surgery or radiation. Tumor severity was classified by International Federation of Gynecology and Obstetrics (FIGO) stages I (least severe) to IV (most severe). FIGO stages are further divided into A–C to reflect tumor spread, resulting in (e.g.) FIGO stage IVB. Data preparation included clearing doublets for patients with several surgeries; the first surgery date (often primary surgery) was kept. Additionally, 20 patients who moved abroad were excluded due to unresolved lifelines. In summary, 1924 ovarian cancer patients, 511 cervical cancer patients, and 2046 corpus cancer patients were included.

#### Data visualization using Lexis diagram

Lexis diagrams of survival data were plotted in R using the Epi package [31]. Dates of diagnosis, surgery, relapse, and death were plotted using lifelines. Different-colored lines and different-colored and -shaped events happening during the patients’ lifetime were used to enhance visualization of care complexity represented by (e.g.) cause of death. It was important to include all patients, regardless of survival, to avoid bias.

#### Data analysis

Lexis diagrams were presented to process owners at each dialogue meeting, visualized according to expected needs from the last meeting. The process owners analyzed data as they saw fit in order to find relevant patterns. This included eye-balling the graph for overall trends, counting dots and lines, or analyzing sections of the diagram. It was clarified to process owners that the patterns may indicate trends but that this does not imply statistical significance. The scope of relevant data was revised based on insights gained during analysis, leading to further updates of diagrams between meetings. Each meeting ended with an action plan for the next step of visualization; this iteration continued until process owners felt that visualizations reflected the important factors for their diagnoses in an understandable way; there were five dialogue meetings in total. Perceived ease of use and perceived usefulness to support QI are important factors for new QI methods, since both reflect behavioral intention to use the method [44]. Therefore, questions regarding usefulness and ease of use were included in the dialogues. Focus of discussions otherwise lay on clinical interpretation, connection to QI, and graphical representation.

## Results

Sample diagrams are presented to illustrate key results and different features and usage across the three cancers, to show different ways to use the diagrams depending on data relevance, with emphasis on feedback, timeliness, and visualizing complexity.

### Ovarian cancer: presenting data in survival curves and Lexis diagrams, respectively

**Fig. 3:**
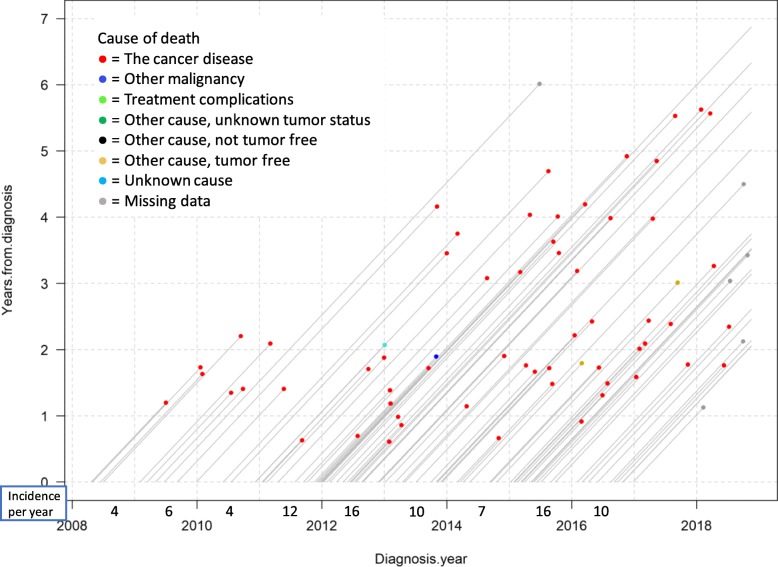
A Lexis diagram of the patients with ovarian and tubular cancer FIGO stages III–IV, diagnosed in years 2008–2016, undergoing delayed surgery. Incidence per year is equal to the number of patients in the cohort each year

To support understanding, Fig. 3 present a Lexis diagram including a small sample size and focusing on cause of death only. The data are survival data for ovarian and tubular cancer FIGO stages III–IV in patients diagnosed from 2008-01-01 to 2016-12-31 that underwent delayed surgery. The patients were followed until death or data extraction day 2018-11-17. Each line represents a patient, entering the diagram on the x-axis on the date of diagnosis, then follows along the line until either death (line ending with a dot) or extraction date (line stretching to the right border). Each dot represents one death, and the different colors of death events represent causes of death, as presented in the legend. The incidence per year for this patient group is presented below the x-axis. From eye-balling the data, there seem not to be any clear trends regarding survival. However, one important and positive conclusion is that this cohort has no treatment-complication-related deaths (which would have been represented by light green dots).

**Fig. 4:**
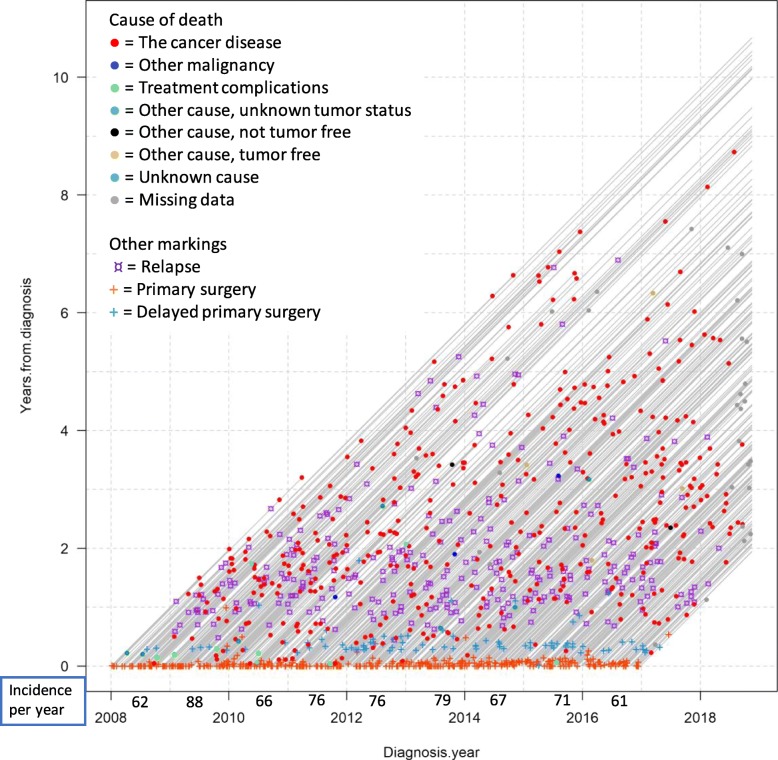
All patients diagnosed with ovarian and tubular cancer FIGO stages III–IV, 2008–2016. Incidence per year is equal to the number of patients in the cohort each year. Note that the visualization is better displayed on the computer screen, see online version

To build up the understanding of complexity, Fig. 4 show data on all ovarian and tubular cancer FIGO stages III–IV patients that underwent surgery, diagnosed from 2008-01-01 to 2016-12-31. The markings show some of the additional information that can be included when individual lifelines are plotted.

As above, the color of the end-line dot for each patient symbolizes cause of death, while the ¤ mark represents relapse date and the cross represents surgery date (see figure legend). The separation between primary surgery and delayed primary surgery was chosen because of different care processes: delayed surgery is preceded by chemotherapy to shrink the tumor before surgery, while in direct primary surgery, chemotherapy is given post-surgery. These subgroups are here presented together, but could also have been presented in separate Lexis diagrams, as in Fig. 3. Noteworthy, some of the same data (diagnosis date 2008-01-01–2013-12-31) have previously been used by the process owners in survival curve analysis form, as published in Dahm-Kähler et al. [15], to evaluate centralizing surgery from regional hospitals to the main university hospital in Region Västra Götaland, as of January 2011 (see Fig. 1a). That data analysis contributed to “global knowledge” on medical improvement by showing significantly lower mortality following centralization. Although survival curves and Lexis diagrams have different purposes, process owners are accustomed to analyzing survival data using survival curves and thus initially compared the two methods with each other, as seen in some of the following quotes.

#### Feedback and timeliness

The ability to continuously add new patients is an advantage of Lexis diagrams. One process owner gave the following first impression regarding gaining feedback from following the care process over time:

*“It [the Lexis diagram] is continuous, enabling closer to real-time monitoring… It is very useful since it is very illustrative…. One sees how the deaths are spread over the years and one sees when the relapses are coming. So, it is a very efficient way to illustrate [survival data]…. One gets faster feedback than waiting for five-years survival analysis.”*

*– Process owner 1*

Since different interventions have been made continuously in the care process, potential root causes need further attention, but Lexis diagrams opened up discussions around improvement efforts, future events, and how to proceed with investigations. For example, counting light green dots revealed that in the cohort diagnosed during the first 4 years, 2008–2011, there were five deaths due to treatment, but only one in the cohort diagnosed in 2012 and onwards, covering 5 years. As noted by a process owner, increase in green dots in Fig. 4 during real-time follow-up would represent a timely alarm that there is a problem with the care process. Generally, Lexis diagrams do not rely on any specific rules or thresholds for when to act; rather, it is driven by domain expertise. As treatment-related death should preferably never happen, a single green dot could be enough to trigger further investigation and action. Counting dots in Fig. 4 is somewhat difficult, and to facilitate feedback on treatment-related deaths (that is, the counting of light green dots), a Lexis diagram presenting only lifelines and cause of death dots, as in Fig. 3, could be produced.

Visualizing care complexity also highlighted potential problems and raised questions concerning data quality, specifically the trustworthiness of the light green dots in Fig. 4. Cause of death is sometimes hard to define, an example being heart failure, which may be a consequence of the very tough cancer treatment, in turn a consequence of the cancer disease; any of these causes of death (heart failure, treatment-related death, death due to cancer) may have been chosen as the cause of death by the pronouncing physician. Treatment-related deaths seemed to happen close to surgery, but since 2014 (see Fig. 4), there have been very few deaths close to surgery, indicating a low risk of treatment-related deaths being registered as any other cause of death. This discussion highlights the potential of Lexis diagrams to drive data quality improvements.

Another aspect of feedback highlighted as important was visualization of surviving patients. Lexis diagrams like the ones in Figs. 3 and 4 present surviving patients too, represented by the lifelines stretching until the extraction date. This use of Lexis diagram visualization for motivation was reflected upon by two process owners:

*“There are rather many patients alive then [after 8–10 years].”*

*– Process owner 2*

*“Yes, and this is still for FIGO stages III and IV.… They are many. This [visualization] I would like to show our resident physicians [who often] lose faith and believe that everyone dies.”*

*– Process owner 3*

*“Exactly! I need to remind our specialist physicians about that also sometimes.… Because you meet those that die, those are the ones you remember and work with, isn’t it? Those [surviving] being home, you never meet again.”*

*– Process owner 2*

#### Complexity

Process owners perceived the options to color both lines and markings as highly useful as they allowed considerable information to be comprehensibly included. The use of Lexis diagrams for visualizing care complexity was contrasted with the use of survival curves by one process owner, as survival curves represent the primary method they use:

*“They are a rather blunt tool, the survival curves, because it shows patients on group level all the time and who is it then that we lose, since they die? We only get information about survivals, so to say. And then one realizes that the other ones have died. And is that the old patients, or who is it? Can one find subgroups from which we can learn how to take better care of them? …. [Presenting more factors in Lexis diagrams] may lead to a hypothesis which needs to be tested in a larger sample [using e.g. survival curves]. But here at least we see that something is happening.”*

*– Process owner 4*

Another process owner reflected on the different purposes and complementary use of survival curves and Lexis diagrams, with regards to the explored purpose of evaluating improvement efforts:

*“This is another thing [than survival curves]…it is another way of thinking if we want to see that we made significant difference. Then we need to use the statistical methods. But to see if one is on the right or wrong track, that indication is maybe possible to get along the way a bit earlier since one can visualize the process. First [with Lexis diagram], we can see if we are on the right track, then, [with statistical methods] we can prove that we are on the right track.”*

*– Process owner 1*

Thus, the findings verify that Lexis diagrams cannot replace survival curves, but can serve as a complement to give stakeholders feedback before statistical analysis.

Another result on visualization of care complexity is that the diagrams were perceived as somewhat unpedagogical, despite considerable work invested by the researcher in finding good contrasts between colors and markings. New users may need introductory education to work with the diagrams, but after that, including several factors should be no problem. In addition, when reducing the diagram to only include causes of death, process owners missed additional information from relapses, etc.; when the basic concept of the Lexis diagram is understood, the “messiness” may be bearable if the complexity visualized in the diagram is understandable.

### Cervical cancer: using age as a time axis

One strength of Lexis diagrams is to present data along several time axes. In this example, age is used on an additional time axis to diagnosis date time axis.

#### Timeliness

**Fig. 5:**
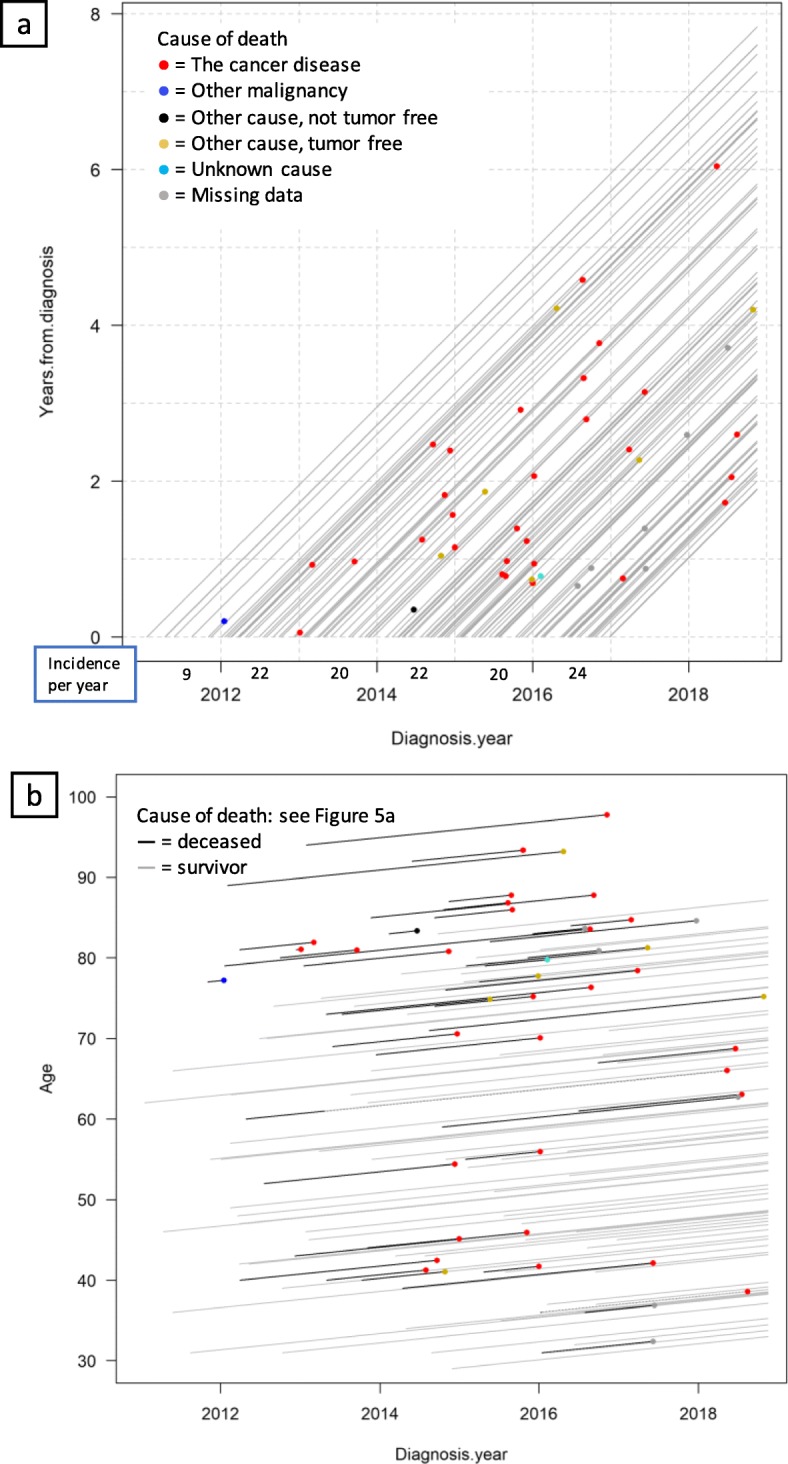
Lifelines for patients with cervical cancer Figo stages IB2–IIB. 5**a**. Lexis diagram showing diagnosis year vs years from diagnosis. Incidence per year is equal to the number of patients in the cohort each year. 5**b**. Lexis diagram showing diagnosis year vs. patient age, with the same dataset and legends as 5**a**

Cervical cancer is rare in Sweden owing to successful screening [45]. This is reflected in Fig. 5a, where only about 20 patients were diagnosed with FIGO stage IB2–IIB a year and even fewer died. This means it takes years to collect enough survival data to statistically analyze effects of care for subgroups, meaning long lead times for feedback on care processes through survival curves.

#### Feedback by following data over time

Age is an interesting factor for cervical cancer patients. Older patients are often fragile, and the treatment is very aggressive. To understand the relation between survival length and patient age, another Lexis diagram was plotted, with patient age on the y-axis instead of years since diagnosis; see Fig. 5b. Contradicting the process owner’s perceived clinical experience, it appeared that age seemed to have had little to do with the deceased’s length of survival in this dataset; indeed, two of three patients who were 90 or older survived for several years and one of them died tumor free from another cause. As with any trend identified, however, such relations need to be tested using proper statistical principles [46] before sound conclusions can be drawn.

#### Complexity

Further, showing patient age on one time axis with causes of death resulted in discussion regarding treatment-related deaths, quality of life, and patient centeredness:

*“It is almost a philosophical question—what is best to die from? Heart failure due to a tough treatment or to die a few months later because we have not been able to control the cancer?”*

*– Process owner 3*

*“Then she spent seven weeks at the hospital to undergo the treatment, instead of being home.”*

*– Process owner 4*

*“Then comes the question what the patient wants as well. Most [patients] say that I know it is a tough treatment but try to remove the cancer. But if they knew that they would die because they had so much nuisance, they might have said something else if one would rewind some months.”*

*– Process owner 3*

*“But this is very important to highlight, how one interprets this data. How one thinks, want to think. How one sorts, consciously, unconsciously.”*

*– Process owner 4*

### Uterine corpus sarcoma: within-diagram stratification of sarcoma types

The idea to visualize all corpus cancer patients stratified in two cohorts by tumor morphology using different line colors had to be abandoned. As over 250 patients are diagnosed with corpus cancer each year and the two cohorts were well mixed over time, the diagram got cluttered and impossible to interpret due to overlapping lines.

**Fig. 6:**
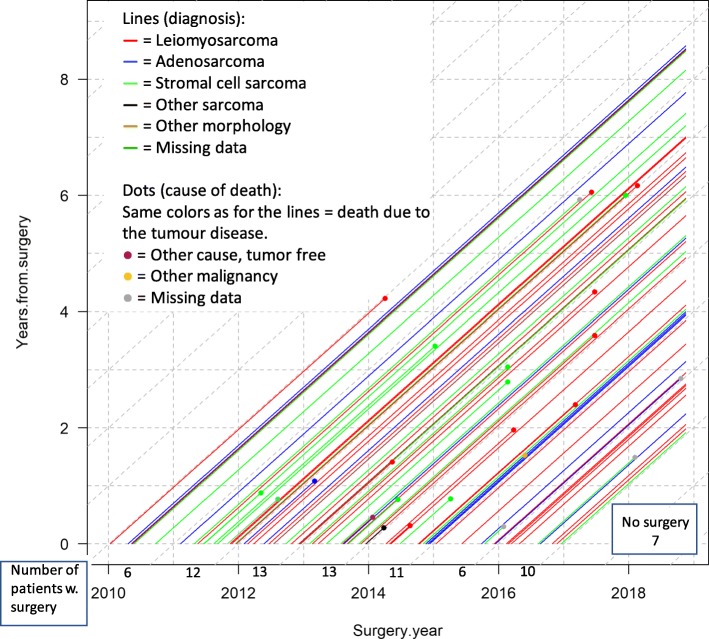
Lexis diagram of uterine corpus sarcomas with lines colored according to sarcoma type. Same-colored lines and dots represent deaths caused by sarcoma; different colored dots and lines represent other causes of death or missing data, see legend. Seven patients diagnosed with uterine corpus sarcomas did not receive surgery. Number of patients w. surgery per year is equal to the number of patients that underwent surgery in the cohort each year

Uterine corpus sarcoma (leiomyosarcoma, adenosarcoma, and stromal cell sarcoma) is a group of rare tumors striking about 10 patients in Western Sweden yearly. Leiomyosarcoma is regarded as the most severe type of uterine corpus sarcomas; to assess this, a Lexis diagram was produced separating the sarcomas through different-colored lifelines. Besides the three main types of sarcomas, the quality registry also considered other sarcoma, other morphology, and no data (missing data); see legend in Fig. 6.

#### Timeliness

Even more than cervical cancer, sarcoma is difficult to analyze statistically due to very low incidence, reaffirming the need to visualize individual patient data for timely feedback on trends, until statistical analysis can be pursued.

#### Feedback by following data over time

Different-colored lines in Fig. 6 show the distribution of different sarcoma types, where the lines or dots representing deaths per sarcoma type were counted. As expected, many deaths are due to leiomyosarcoma, but stromal cell sarcoma seems to kill proportionally more (about half) of patients than leiomyosarcoma (about a third).

#### Complexity

The description of complexity is here focused on highlighting the different sarcoma types and identification of the patients with tumor disease as cause of death. This is made possible by highlighting these patients using same-colored dots and lines, thus easily differentiating the deaths caused by the different sarcomas. Additionally, a few patients died from other causes of death highlighted by different coloring between line and dot, such as one patient with leiomyosarcoma who died tumor free from another cause (red line, purple dot). Moreover, missing cause of death (grey dot) highlights data quality issues.

## Discussion

Dialogue with process owners indicated that Lexis diagrams are useful for survival data analysis. Process owners highlighted the potential of Lexis diagrams for both individual and population-level data [33], including using the visualization to build testable hypotheses [8]. Further, feedback by following data over time, timeliness, and complexity were recognized as prominent features in QI for survival data.

### Feedback by following data over time and timeliness

As new patients, patient events, and attribute data can be added in real time, Lexis diagrams enable continuous monitoring of and feedback on care processes, which are central to QI [47]. However, as Lexis diagrams show trends rather than significant changes, process owners saw the results only as early indicators and real-time feedback for timely evaluation of care (which may support hypothesis-testing using statistical analysis), which they saw as valuable despite its lack of statistical significance. Earlier research confirms the importance of feedback [12, 48] in QI. Data visualization seemed to be an important driver, as some analysis, such as deaths due to sarcomas, could have been analyzed in tabular form also, but data visualization was still preferred by most process owners, reflecting the power of visualization [49].

### Complexity

The diagrams’ ability to capture some of the complexity of care was appreciated, and using different perspectives for the three sub-diagnoses revealed the diagrams’ versatility. Building up complexity iteratively as process owners learned more about the Lexis diagrams, rather than including all complexity initially, probably helped learning [50]. The possibility of using different markings and colors for lifelines and attribute data or changing time axes to enhance visualization was useful to identify relevant patterns. Still, only relevant factors should be included, to avoid cluttering the diagram. This reflects QI theory, which recommends simple graphical illustrations to aid understanding [4], and visualization research, which argues for visualizations requiring minimal effort to interpret, to enable sensemaking [8]. Note also that complexity and understanding data are to some degree antipodean [17]: the more complexity is included, the less the chance of understanding the data. Therefore, a balance between level of aspiration of understanding and complexity is needed, driven by domain expertise. Basic Lexis diagrams are mainly useful for small data sets [30], and large data sets should preferably be analyzed using more advanced Lexis diagrams [25, 26, 34].

### Additional insights on usefulness

#### The patient perspective

Visualization of individual patient lifelines increased patient focus, which is important to QI [51], by allowing process owners to understand individual complexity. They moved beyond the narrow focus on survival or death to see the patients behind the data and their quality of life. Why did they die? What happened? How do they want to spend their last time? Moreover, process owners saw potential to motivate co-workers through increasing focus on the many surviving patients, instead of focusing on the (generally) few that die. Earlier research confirms positive effects of data feedback to increase motivation, provided the data are seen as trustworthy [52].

Summarizing its perceived usefulness, the Lexis diagram was not only useful to analyze data but also to present data to decision-makers in care organizations to support continuation or change of current practice and to motivate colleagues. Inclusion of Lexis diagrams within Swedish care organizations would be possible through the existing platform in which regional patient data are continuously updated and presented in various ways. Lexis diagrams can be analyzed quarterly, which is how frequently process owners normally use the platform for data feedback.

#### Major drawbacks

It became apparent that caution is needed in incorporating additional factors in the diagrams. As individual patient data are plotted, case-mix adjustment is not an option; therefore, caution is needed when interpreting data, so that differences in (e.g.) number of dots each year are related to lifeline density, in this study equal to the incidence rate of the disease. One potential drawback with the basic Lexis diagram is the possibility that intermediate events, such as relapse, may affect how survival or the duration of the disease is dependent on time [53]. In such a case, an additional time axis in the Lexis diagram could be used, rendering a 3D visualization [54].

Although process owners appreciated the option of adding different markings and colors for attribute data, too much information cluttered the diagram, making it difficult or even impossible to interpret. This highlights the need to show only relevant data [21], which may be supported by interactive visualization, as exemplified in Shneiderman et al. [55].

### Limitations and future research

This study has some limitations. The survival data cover only one type of diagnosis, in one part of Sweden, and collaboration was conducted with only one group of process owners. Still, by focusing on several sub-diagnoses and analyzing different types of visualizations, a broad view was given of the usefulness of Lexis diagrams. As most insights were on a general QI level rather than specific to gynecological cancer, meaningful insights may result from attention to other contexts as well.

As different types of Lexis diagrams and attribute data were shown to be useful in different contexts and too much information cluttered the visualization, future research may strive to develop interactive Lexis diagram platforms, enabling attribute data to be chosen depending on need and helping viewers understand the relatedness of clinically relevant attribute datapoints [21]. These platforms could be extended to include 3D visualization as well, to address secular time trends [54].

Although the researcher constructed all visualizations while the process owners contributed with domain knowledge, the iterative analysis increased the method understanding among process owners over time. Therefore, the process owners may have been somewhat biased in their interpretation of the method’s usefulness towards the end as compared to future novel users, and it should be noted that education and experience may be needed to fully grasp the method, preferably using an interactive version of the Lexis diagram. Future research on Lexis diagram usefulness is encouraged also to present Lexis diagrams to practitioners not included in the construction of the diagrams and gain insights about their perceptions of Lexis diagram usefulness.

Finally, Lexis diagrams are not limited to survival data but may be used for following any care process, just like survival curves [16]. Future research can address the use of Lexis diagrams for other diseases where feedback by following data over time, timeliness, and complexity of care are meaningful analytical criteria.

## Conclusion

This study shows that Lexis diagrams can support QI, through survival data analysis. By enabling continuous, close-to-real-time data updating, Lexis diagrams were shown to support timeliness, which is key to relevant action for QI. Feedback, another important aspect of QI, was enabled by following data over time, allowing understanding of trends and hypothesis-building based on changes to the process. However, unlike Lexis diagrams, survival curves include statistical significance testing, and may therefore complement Lexis diagrams for hypothesis-testing. Further, this study shows the importance of analyzing the complexity of care processes. Visualizing attribute data supports practitioners’ understanding of the care process, facilitating communication and triggering important dialogues between practitioners and thereby supporting evaluation of given care or identification of future QI efforts. However, since too much data clutters visualization, confusing rather than supporting understanding, it is important to include only relevant data, presented in a clear way. The versatility of Lexis diagrams, through different time axes, coloring, and markings, was shown to be useful to address different questions and analyze different perspectives on the care process.

One positive affordance of Lexis diagrams, linked to both feedback and complexity, is the combined visualization of individual and population levels. The ability to see the individual patient as part of a population not only supported understanding of the care process but also facilitated increased focus on the patient’s needs. Shifting focus from deceased patients to include survivors may further affect motivation among healthcare practitioners and management. The Lexis diagram thereby has a non-overlapping potential application compared to survival curves.

Care is needed, however, when using Lexis diagrams, so that false conclusions are avoided, whether stemming from bias toward a sub-group of patients, possible variation in incidence rate, or visualizing data of questionable quality. These aspects may be addressed by educating new users and reflecting on how data are presented in Lexis diagrams, through analyzing visualizations in close collaboration with care process experts.

## Data Availability

The data analyzed during this study are held by RCC West, Sweden after completed ethical trials, and are thus available upon request to RCC West and not from the corresponding author. R codes are available from the corresponding author on reasonable request.

## Abbreviations

FIGO: Staging system for cancer by International Federation of Gynecology and Obstetrics
QI: Quality improvement
RCC: Regional Cancer Centre
